# Supplementary material for: Heterologous Expression and Characterization of Plant Wax Ester Producing Enzymes
Source: Metabolites. 2022 Jun 22;12(7):577. doi: 10.3390/metabo12070577 (PMC9319179; doi:10.3390/metabo12070577)
Supplement: Supplementary file 1 [file metabolites-12-00577-s001.zip › Figure S1.BoxPlot.pdf]

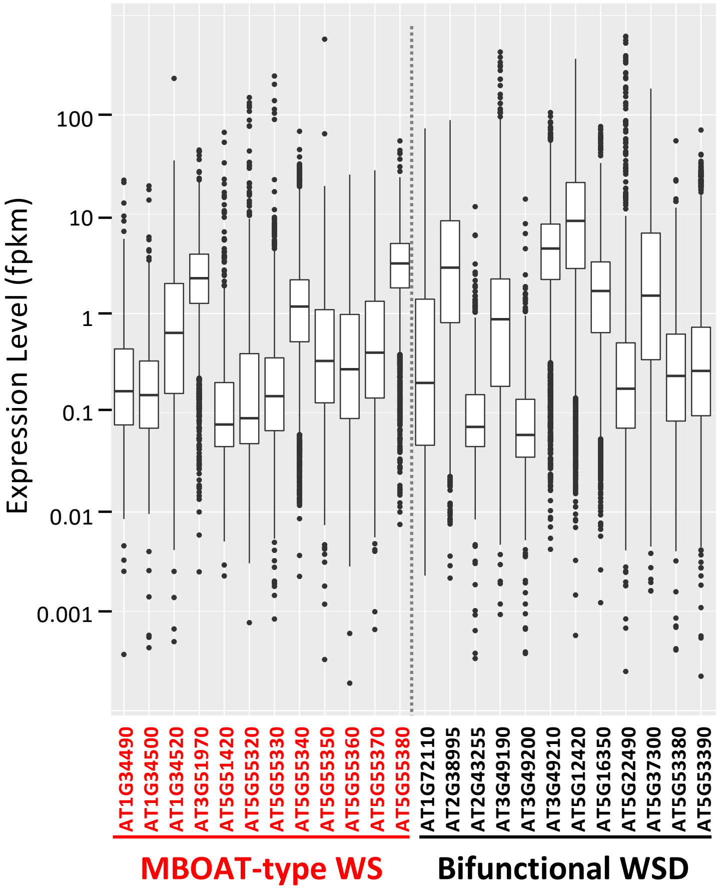

Figure S1. Box-plot presentation of the range of expression levels of the 12 WS genes and 11 WSD genes determined from 5200 individual Arabidopsis RNA-Seq experiments downloaded from the NCBI Sequence Read Archive (Table S3).
