# Supplementary material for: Heterologous Expression and Characterization of Plant Wax Ester Producing Enzymes
Source: Metabolites. 2022 Jun 22;12(7):577. doi: 10.3390/metabo12070577 (PMC9319179; doi:10.3390/metabo12070577)
Supplement: Supplementary file 1 [file metabolites-12-00577-s001.zip › Figure S2.Ling.ConstructMaps_Supplemental .pdf]

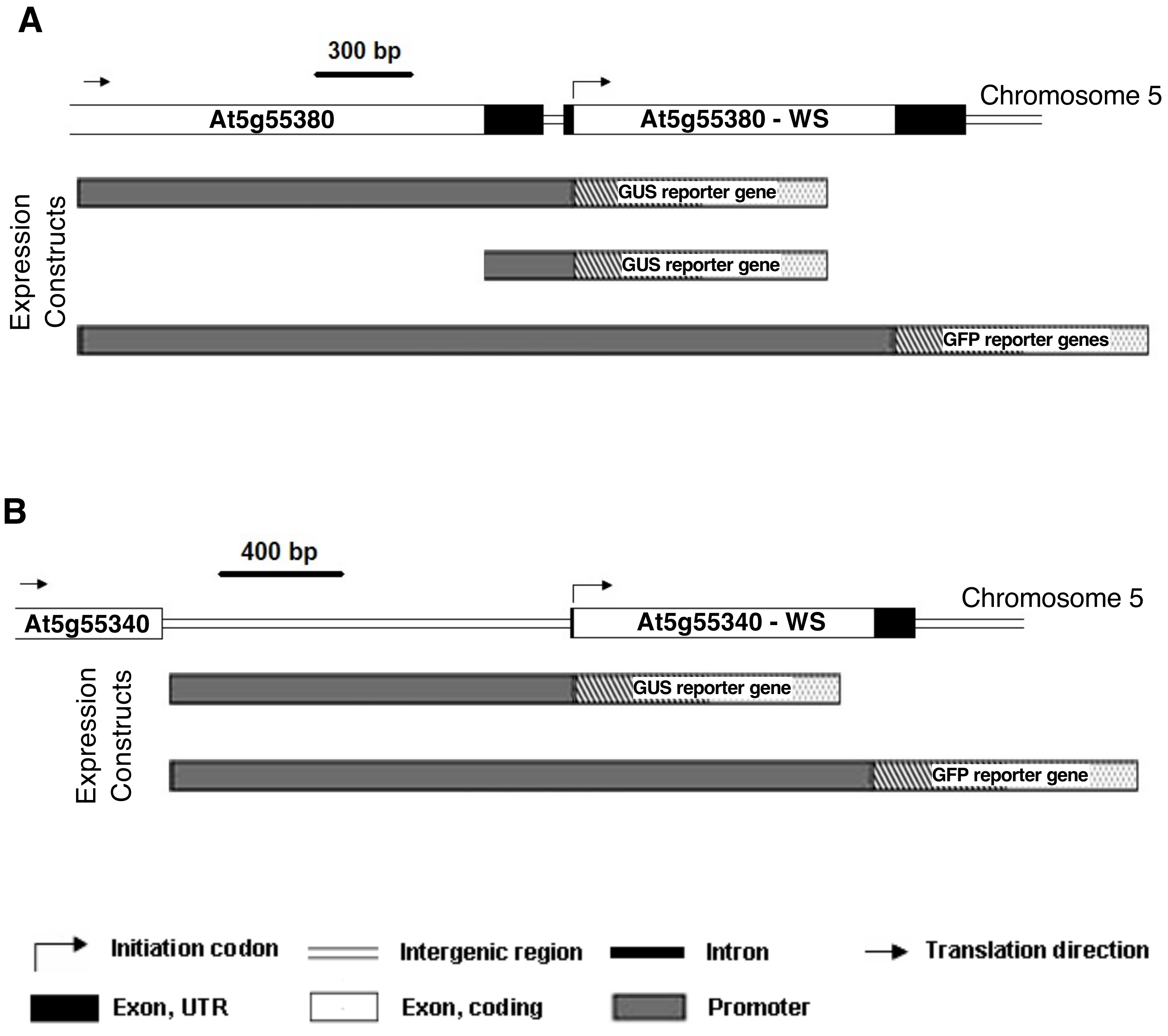

Figure S2. Wax synthase reporter transgene constructs.

(A) Assembly of GUS or GFP expression constructs for the At5g55380 WS gene, using genomic DNA fragments isolated following PCR amplification.

(B) Assembly of GUS or GFP expression constructs for the At5g55340 WS gene, using genomic DNA fragments isolated following PCR amplification.
